# Copy Number Variation of Mitochondrial DNA Genes in *Pneumocystis jirovecii* According to the Fungal Load in BAL Specimens

**DOI:** 10.3389/fmicb.2016.01413

**Published:** 2016-09-12

**Authors:** Clara Valero, María José Buitrago, Maud Gits-Muselli, Marion Benazra, Aude Sturny-Leclère, Samia Hamane, Nicolas Guigue, Stéphane Bretagne, Alexandre Alanio

**Affiliations:** ^1^Servicio de Micología, Centro Nacional de Microbiología, Instituto de Salud Carlos IIIMadrid, Spain; ^2^Laboratoire de Parasitologie-Mycologie, Groupe Hospitalier Saint-Louis–Lariboisière–Fernand-Widal, Assistance Publique – Hôpitaux de ParisParis, France; ^3^Sorbonne Paris Cité, Université Paris DiderotParis, France; ^4^Institut Pasteur, Unité de Mycologie Moléculaire, Centre National de Référence Mycoses Invasives et Antifongiques, Institut PasteurParis, France; ^5^CNRS URA3012Paris, France

**Keywords:** *Pneumocystis jirovecii*, real-time PCR, DNA quantification, PcP, carriage, mitochondria, copy number variation

## Abstract

*Pneumocystis jirovecii* is an unculturable fungus and the causative agent of *Pneumocystis* pneumonia, a life-threatening opportunistic infection. Although molecular diagnosis is often based on the detection of *mtLSU rRNA* mitochondrial gene, the number of copies of mitochondrial genes had not been investigated. We developed and optimized six real-time PCR assays in order to determine the copy number of four mitochondrial genes (*mtSSU rRNA*, *mtLSU rRNA*, *NAD1*, and *CYTB*) in comparison to nuclear genome (*DHPS* and *HSP70*) and tested 84 bronchoalveolar fluids of patients at different stages of the infection. Unexpectedly, we found that copy number of mitochondrial genes varied from gene to gene with *mtSSU rRNA* gene being more represented (37 copies) than *NAD1* (23 copies), *mtLSU rRNA* (15 copies) and *CYTB* (6 copies) genes compared to nuclear genome. Hierarchical clustering analysis (HCA) allowed us to define five major clusters, significantly associated with fungal load (*p* = 0.029), in which copy number of mitochondrial genes was significantly different among them. More importantly, copy number of *mtLSU rRNA*, *NAD1*, and *CYTB* but not *mtSSU rRNA* differed according to *P. jirovecii* physiological state with a decreased number of copies when the fungal load is low. This suggests the existence of a mixture of various subspecies of mtDNA that can harbor different amplification rates. Overall, we revealed here an unexpected variability of *P. jirovecii* mtDNA copy number that fluctuates according to *P. jirovecii*’s physiological state, except for mtSSU that is the most stable and the most present mitochondrial gene.

## Introduction

*Pneumocystis jirovecii* is an ascomycetous fungus that is specifically associated to human lung microbiota (Cushion, 2010; Gigliotti and Wright, 2012). *P. jirovecii* thrives at the surface of alveolar pneumocytes in humans but fails to grow on artificial media. However, air-liquid interface culture system have been developed with demonstration of *P. jirovecii* amplification *in vitro* (Schildgen et al., 2014). These characteristics have made difficult to study its genetic diversity, complexity and evolution in humans. In particular, the nuclear genome of *P. jirovecii* has only recently been sequenced (Cissé et al., 2012; Cushion and Keely, 2013) and its mitochondrial genome recently described (Ma et al., 2013). It is now well accepted that *P. jirovecii* circulates within normal hosts with interhuman transmission through air (Choukri et al., 2010; Cushion, 2010; Gigliotti and Wright, 2012). Primary infection occurs very early in life with almost all infants being exposed to *P. jirovecii* before the age of 2 years based on serological surveys and detection of *P. jirovecii* DNA in healthy children (Vargas et al., 2001; Bishop and Kovacs, 2003). The transmission of *P. jirovecii* to immunocompromised host in the absence of prophylaxis results in progressive increase of the fungal burden in lungs (Choukri et al., 2011). Asymptomatic carriage or colonization as defined as detection of *P. jirovecii* DNA in asymptomatic patients is common in immunocompromised population representing about 15–20% of the patients (Alanio et al., 2011; Morris and Norris, 2012; Mühlethaler et al., 2012). If a carrier patient remains immunocompromised, *Pneumocystis* pneumonia (PCP) can occurs within the following weeks (Mori et al., 2009). Indeed, *P. jirovecii* is known to cause PCP, especially in patients with cellular immunosuppression such as HIV-positive, solid organ transplant and cancer/hematology patients, but also in adults and children with other underlying conditions (Pagano et al., 2002; Roblot et al., 2003; Catherinot et al., 2010; Wissmann et al., 2010; Reid et al., 2011; Mori and Sugimoto, 2012; Tasaka and Tokuda, 2012). PCP symptoms have been described more severe and death rates have been considered significantly higher in HIV-negative in comparison to HIV-positive patients (Roux et al., 2014).

Historically, the diagnosis of PCP relied only on the visualization of the fungal forms (trophic forms) and asci (cysts) using classical staining (Giemsa, Gomori methenamine silver, Toluidine Blue, Calcofluor) or direct or indirect immunofluorescence stainings (Alanio et al., 2016b). These methods lack sensitivity and specificity and need microscopical expertise compared to PCR methods that have been developed since the 1990’s (Wakefield et al., 1990). In the past 15 years, *P. jirovecii* DNA amplification assays have emerged as new diagnostic tools for PCP diagnosis especially when real-time quantitative PCR (RT-PCR) has been used as the most reliable method for diagnostic PCR assays (Alanio et al., 2016b). Quantitative results are of prime interest since carrier patients can be detected for *P. jirovecii* DNA as patients with PCP. Indeed, thresholds with a gray zone have been proposed to classify patients in terms of probability of having PCP (Flori et al., 2004; Alanio et al., 2011; Mühlethaler et al., 2012). To increase sensitivity, repeated targets have been selected with the mitochondrial large ribosomal subunit (*mtLSU rRNA*) as the main target used in diagnostic assay. It has been observed by using electron microscopy that each trophic form carried one mitochondria but the number of mitochondrial DNA (mtDNA) copies per organism have not been clearly investigated. According to some reports, mtDNA of *P. jirovecii* may have circular configuration in contrast to closely related species *P. carini* and *P. murina*, in which mtDNA has a linear conformation. Despite this change in configuration, all species share the same set of genes but in a different ordering (Ma et al., 2013). However, the physiology of the mitochondria in *P. jirovecii* is mostly unknown, which makes difficult to rely *P. jirovecii* quantification only on mitochondrial genes.

The aim of this study was to analyze the quantification of four mitochondrial genes located in different places of the mitochondrial genome in comparison to two nuclear unique genes in respiratory samples of patients harboring various clinical situations.

## Materials and Methods

### Ethics Statement

Saint-Louis Hospital, Paris, France, is a 650-bed tertiary university hospital with main clinical activities in hematology and oncology. This study was a retrospective non-interventional study. Biological material and clinical data were obtained only for standard diagnostic procedures following physicians’ prescriptions with no specific sampling. According to the French Health Public Law (CSP Art L1121-1.1), such study did not require approval of an ethics committee and is exempted from specific informed consent application.

### Patients and Clinical Samples

All *P. jirovecii* PCR positive clinical samples containing more than 10 trophic form equivalents/mL, according to the quantification method based on the qPCR assay proposed by Alanio et al. (2011), were selected for this study. These samples were collected from patients managed in Saint-Louis Hospital, Paris, France, and processed and stored at -20°C in our laboratory. DNA extraction of the clinical samples was performed by using a QIAamp DNA Mini kit (Qiagen, Hilden, Germany), according to the manufacturer’s instructions as already described (Alanio et al., 2011).

A total of 86 bronchoalveolar lavage (BAL) fluids from 84 patients were selected. Clinical (background of the patient, the final diagnosis of the infectious episode) and biological (immunofluorescence results, PCR quantification) parameters were collected retrospectively from electronic patient files and from our biological data management software, respectively. For all analysis, only the first sample per patient was considered in the analysis to avoid redundancy in the data.

A second cohort of 95 *mtLSU rRNA* PCR-negative samples from the routine testing data was tested for the presence of *mtSSU rRNA* amplification.

### Real-Time Quantitative PCR Assays

Primers and probes were designed to amplify six different *loci* of *P. jirovecii* genome using Primer3web v4.0.0 software. Four of them (*mtSSU rRNA, mtLSU rRNA*, *NAD1*, and *CYTB*) were mitochondrial genes, and two (*DHPS* and *HSP70*) were unique nuclear genes. Sequences of primers and probes used in this study and the size of the amplicon generated, are detailed in **Supplementary Table S1**. RT-PCR assays were carried out in a LightCycler 480 unit (Roche Diagnostics, Mannheim, Germany). PCR reactions were performed in 25 μl-final volume containing 0.3 μM of each pair of primers and 0.1 μM of probe for each target tested in the assay. For *mtLSU*, *DHPS*, *NAD1*, and *mtSSU* quantification assays, 2x LightCycler 480 Probes Master (Roche Diagnostics, Mannheim, Germany) was used, whereas 2x TaqMan Universal PCR Master Mix (Applied Biosystems, Foster City, CA, USA) was added for *CYTB* and *HSP70* quantification analysis. Finally, 5 μl of DNA extracted from clinical samples were added in duplicate to each quantification assay. PCR conditions were as follows: an initial step of 10 min at 95°C, following by 45 cycles at 95°C for 15 s and 60°C for 30 s with an ending cooling phase of 30 s at 40°C. Results were considered positive when the fluorescent signal above the baseline was detected, as determined by second-derivate analysis and were expressed in terms of the quantification cycle (Cq). Each experiment included a positive control consisted in a dilution of DNA extracted from a controlled clinical sample positive for PCP at high fungal load as well as negative controls.

### Standardization and Data Analysis

A standard curve for each genomic target allowing PCR efficiency calculation was obtained based on the result of two PCR repetitions with five 10-fold serial dilutions of a controlled DNA extracted from a clinical sample positive for PCP at high fungal load and previously quantified by RT-PCR (Alanio et al., 2011). Regression lines were constructed automatically by plotting the logarithm of the initial template concentration versus the corresponding Cq value by using Analysis package included in LightCycler 480 software v. 1.5 (Roche Diagnostics, Mannheim, Germany). For the calculation of the copy number, the minimal Cq value obtained was selected from the duplicates values. The Cq ratio between each mitochondrial gene and the geometric mean of the two monocopy nuclear genes was calculated as described by Pfaffl (2001) and Vandesompele et al. (2002). The geometric mean of two monocopy nuclear genes was considered as the best value representing the nuclear genome and called “nuclear genes” along the manuscript.

Finally, a specificity test was performed by testing a panel of different fungal DNAs at 0.01 ng/μL for each target analyzed in this work. DNAs tested belonged to fungal species present, normally, in the human respiratory tract: *Aspergillus fumigatus* Af293, *A. niger* CNRMA15.743, *Rhizopus microsporus* CNRMA14.351, *Mucor circinelloides* CNRMA16.241, *Candida albicans* CNRMA16.291, *C. glabrata* CNRMA16.324, *Cryptococcus neoformans* CNRMA16.024, *Trichosporon asahii* CBS2479, *Malassezia furfur* CNRMA15.762, identified at the French National Reference Center for Invasive Mycoses and Antifungals.

### Statistical and Graph Analysis

Physical mapping of the *P. jirovecii* mitochondrial DNA was generated in Geneious software v.8.1.5. based on the JX499143 sequence provided by Ma et al. (2013).

Box-and-whisker plots with minimum-maximum were used for data representation. For comparisons, we performed unpaired *t*-test and ANOVA analysis for normally distributed data and Mann–Whitney and Kruskal–Wallis tests for data that were not normally distributed, and χ^2^ test and Fisher’s exact test for contingency tables analyses. Median and interquartile ranges are described in the text. *P*-values of <0.05 were considered significant and the *p*-value was indicated by asterisks as follows: ^∗^*p* ≤ 0.05, ^∗∗^*p* ≤ 0.01, ^∗∗∗^*p* ≤ 0.001, ^∗∗∗∗^*p* ≤ 0.0001. Both graphs and statistical analysis were performed by using Prism 6.0 (GraphPad Software Inc., La Jolla, CA, USA). Hierarchical clustering analysis (HCA) of samples was performed by using TIGR Multiexperiment Viewer (MeV) software v4.6.1 (Saeed et al., 2003).

## Results

### Six Real-Time Quantitative PCR Assay for Determining Mitochondrial DNA Copy Number Variation (CNV)

Copy number of mitochondrial genes (*mtLSU*, *mtSSU*, *NAD1*, and *CYTB*, **Supplementary Figure S1**) was calculated using qPCR. All the six qPCR assays were optimized (**Supplementary Figures S2**–**S4**). A positive control DNA stored at -20°C as 12 μL aliquots was run to validate each run. PCR efficiencies calculated based on 10-fold serial dilutions of the positive control DNA were as follows: *mtLSU rRNA*, *E* = 1.938; *CYTB*, *E* = 1.97; *NAD1*, *E* = 1.968; *mtSSU rRNA*, *E* = 1.931; *DHPS*, *E* = 1.932 and *HSP70*, *E* = 1.95 (**Supplementary Figure S5**). Minimum Cq results of the six genes were used to calculate the ratio between each mitochondrial gene and the geometric mean of the two nuclear genes. The mean Cq value of the positive control DNA included in each PCR assay was 29.18 ± 0.3 for *mtSSU rRNA*, 30.30 ± 0.28 for *mtLSU rRNA*, 29.16 ± 0.16 for *NAD1*, 30.87 ± 0.36 for *CYTB*, 34.69 ± 0.43 for *DHPS* and 35.98 ± 0.58 for *HSP70*.

In order to evaluate the specificity of the assay, DNAs belonging to nine different fungal species were tested for all genes studied in this work. No cross-reactivity to any of the tested fungi was detected.

### The *P. jirovecii* Fungal Load Does Not Reflect the Results of Immunofluorescence and the Final Clinical Diagnosis

According to clinical sample selection criteria explained in section “Materials and Methods” (see Patients and clinical samples), 84 BAL fluids were finally included in the study. The median age of patients was 61 years and the male:female ratio was 1.6:1. Clinical and biological parameters are summarized in **Table 1**.

**Table 1:** Main clinical and biological characteristics of the patients and samples (*n* = 84) included in the study.

	Number of samples (*n* = 84)	*mtLSU* Cq (mean ± SD)
**CLINICAL CHARACTERISTICS**		
**Underlying disease**		
HIV	20	/
Kidney SOT	8	/
Hematology disease	35	/
Others	18	/
na	3	/
**Final clinical diagnosis**		
PCP	44	28.42 ± 4.24
PCC	34	33.75 ± 2.18
na	6	30.87 ± 3.48
**BIOLOGICAL CHARACTERISTICS**		
**Fungal load**		
High (Cq < 30)	29	25.60 ± 2.69
Medium (30 < Cq > 34)	31	32.29 ± 1.20
Low (Cq > 34)	24	35.00 ± 0.72
**IF assay**		
IF+	24	26.33 ± 3.76
IF-	54	33.01 ± 2.56
na	6	28.13 ± 3.94


*HIV, human immunodeficiency virus; SOT, solid organ transplant; PCP, *Pneumocystis* pneumonia; PCC, *Pneumocystis* carriage; IF, immunofluorescence; BAL, bronchoalveolar lavage fluids; na, non-available data.*

**FIGURE 1:**
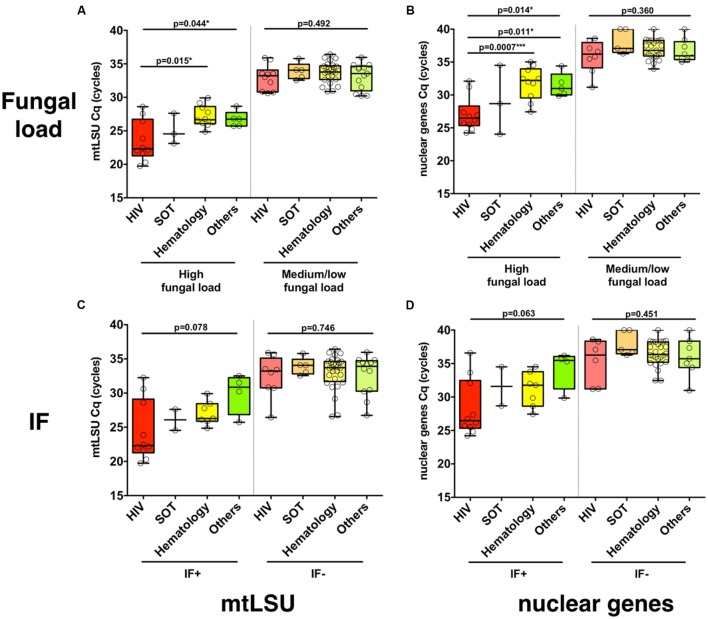
***Pneumocystis jirovecii* fungal load varies according to the clinical background of the patients.** The distribution of *P. jirovecii* quantification in bronchoalveolar lavage fluid (BAL) of patients with different clinical background (HIV positive patients, solid organ transplant patients or other background) is shown for high or medium/low fungal loads **(A,B)** or for immunofluorescence (IF)-positive or -negative BAL **(C,D)** as quantified using *mtLSU rRNA* gene **(A,C)** or nuclear genes **(B,D)**. *P-*values indicating the significance of the differences are indicated in the top of the figure for each category.

In BAL samples with high fungal load, the load was significantly different regarding the background of the patient (HIV, SOT, hematology, others), as determined from *mtLSU rRNA* (*p* = 0.044) and from nuclear genes (*p* = 0.014) quantification with significant differences between HIV and hematology patients (*mtLSU* Cq, *p* = 0.0015; nuclear genes Cq, *p* = 0.0007) and HIV and other backgrounds (nuclear genes, *p* = 0.011) (**Figures 1A,B**), but not with *NAD1* or *CYTB* (**Supplementary Figures S6A,B**). In samples with medium or low fungal load, no differences were observed between the different clinical backgrounds (*p* > 0.05). In contrast, no significant difference (*p* > 0.05) was observed between the different clinical backgrounds in immunofluorescence positive or negative BAL samples for *mtLSU rRNA* (**Figure 1C**) or nuclear genes (**Figure 1D**). The same feature was observed for the final diagnosis classification (PCP vs. PCC) (**Supplementary Figures S6C,D**)

**FIGURE 2:**
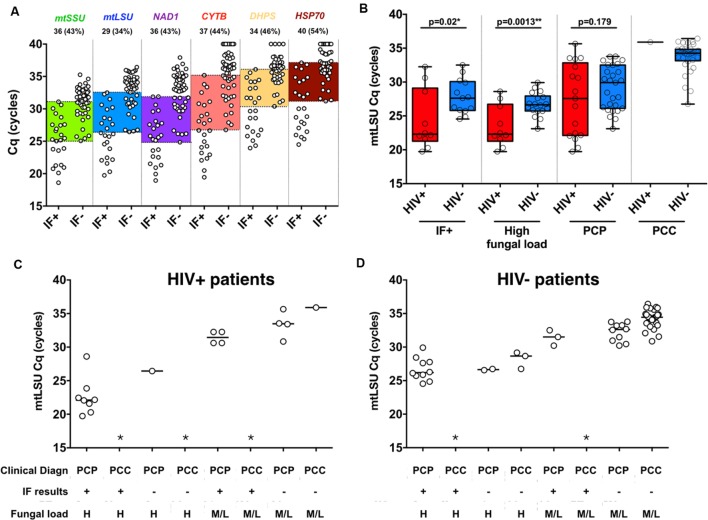
**Immunofluorescence assay and final clinical diagnosis does not reflect *P. jirovecii* fungal load in BAL samples.** Quantification of the six genes included in the study regarding immunofluorescence results of clinical samples **(A)**. Large overlaps between the low IF+ sample and the higher IF- samples are highlighted with color boxes. The number and percentages of samples included in those overlaps are detailed in the top of the figure for each gene. *mtLSU rRNA* gene quantification in HIV+ and HIV- patients for immunofluorescence-positive, high fungal load and final clinical diagnosis **(B)**. *mtLSU rRNA* gene quantification regarding final clinical diagnosis, immunofluorescence results and fungal load in HIV positive **(C)** patients and HIV negative **(D)** patients. ^∗^indicates that this situation was not present in our dataset.

A large overlap of BAL fungal load is observed regarding the results of immunofluorescence in all six genes with 34–54% of the samples included between the last IF+ and the first IF- sample, as highlighted in color (**Figure 2A**). In addition, the BAL fungal load as determined by *mtLSU rRNA* Cq was plotted regarding the result of immunofluorescence and the final diagnosis retained by the clinicians (**Figure 2B**). Large overlaps between PCP and PCC patients are observed in each category either in HIV+ patients (**Figure 2C**) or in HIV- patients (**Figure 2D**) preventing determination of absolute thresholds.

### Mitochondrial Genes Harbor Different Copy Numbers, Which Vary the According to the Fungal Load

**FIGURE 3:**
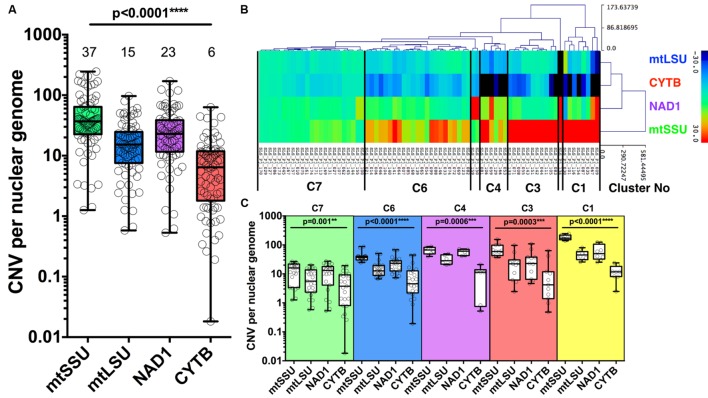
**Copy number variation of four mitochondrial genes.** Copy number variation (CNV) of *P. jirovecii* mitochondrial genes varies from gene to gene with a median of six copies for *CYTB*, 23 for *NAD1*, 15 for *mtLSU*, and 37 for *mtSSU* compared to the mean of two nuclear genes (*DHPS* and *HSP70*) **(A)**. Median copy number is indicated in the top of the figure for each target. Heat map of the copy number of the four mitochondrial genes of the 74 BALs as the result of hierarchical clustering analysis (HCA) **(B)**. Five major clusters containing ≥6 samples were defined as indicated between bars **(B)** with the detailed quantification of each gene within each cluster **(C)**. *P-*values indicating the significance of the differences between each gene in a given cluster and indicated in the top of the figure for each category.

The median number of copies of the four mitochondrial genes varies compared to nuclear genome with *mtSSU*, *mtLSU*, *NAD1*, and *CYTB* harboring a median copy number of 37 [interquartile range, 23–64], 15 [8–25], 23 [12–39], 6 [2–11], compared to the geometric mean of *DHFR*, *HSP70* (nuclear genome), respectively (**Figure 3A**).

Hierarchical clustering analysis of 74 of the 84 BALs based on the variation of copy number of mitochondrial genes from the median value allowed us to define five major clusters (≥6 samples per cluster): cluster 1 (*n* = 8), cluster 3 (*n* = 11), cluster 4 (*n* = 6), cluster 6 (*n* = 23) and cluster 7 (*n* = 23) (**Figure 3B**). Of note, mitochondrial CNV was not calculable for the 10 BALs for which no amplification of the nuclear genes (*DHPS* and *HSP70)* was observed. CNV of all mitochondrial genes were statistically different among all clusters described. However, the difference between the number of copies of the four genes is less important in the 23 samples from cluster 7 (C7) (**Figure 3C**).

**FIGURE 4:**
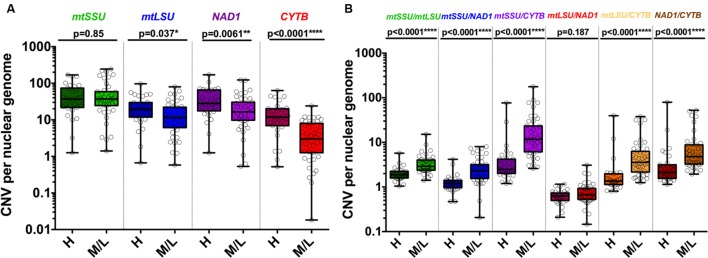
**Copy number variation of mitochondrial genes varies according to the fungal load.** CNV is significantly different in samples with high and medium/low fungal loads for *mtLSU*, *CYTB* and *NAD1* but not for *mtSSU* **(A)**. Each ratio of the DNA of the four mitochondrial genes is significantly different in samples with high and medium/low fungal loads except *mtLSU*/*NAD1* **(B)**. *P-*values indicating the significance of the differences are indicated in the top of the figure for each category.

These five clusters were significantly associated with the fungal load (high vs. medium/low, *p* = 0.029) but not with immunofluorescence (IF+ vs. IF-, 0.11) or clinical status (PCP vs. PCC, *p* = 0.137) (**Table 2**). In details, C1 was significantly associated with medium/low fungal load (0% vs. 18.2%, *p* = 0.020). Significant differences in the CNV between high and medium/low fungal loads were observed for *mtLSU* (*p* = 0.037), *NAD1* (*p* = 0.0061), and *CYTB* (*p* < 0.0001) (**Figure 4A**). The ratio of these genes between high and medium/low fungal loads were significantly different (*p* < 0.0001), except for the *mtLSU*/*NAD1* ratio (*p* = 0.187) (**Figure 4B**). The CNV of *NAD1* and *CYTB* and all the ratios were still significantly different when three categories of fungal load were taken into account (high vs. medium vs. low, **Supplementary Figures S7A,B**). Differences for the *CYTB* gene (**Supplementary Figures S8A,C**) and all ratios for regarding IF results and final clinical diagnosis were significant (*p* < 0.05) except for the *mtLSU*/*NAD1* ratio regarding IF results (**Supplementary Figures S8B,D**).

**Table 2:** Distribution of the samples according to the hierarchical clustering algorithm (HCA) clusters and the fungal load, immunofluorescence or final clinical diagnosis.

	HCA clusters					
	
	C1	C3	C4	C6	C7	*p*^∗^
High fungal load (%) *n* = 27	0 (0)	2 (7.4)	4 (14.8)	9 (33.3)	12 (44.4)	0.029
Medium/low fungal load (%) *n* = 44	8 (18.2)	9 (20.4)	2 (4.5)	14 (31.8)	11 (25)	
IF+ (%) *n* = 23	1 (4.3)	2 (8.7)	2 (8.7)	7 (30.4)	11 (47.8)	0.111
IF- (%) *n* = 42	7 (16.6)	9 (21.4)	4 (9.5)	14 (33.3)	8 (19)	
PCP (%) *n* = 41	2 (4.9)	5 (12.2)	4 (9.8)	14 (34.14)	16 (39)	0.137
PCC (%) *n* = 26	6 (23.1)	5 (19.23)	1 (3.8)	8 (30.8)	6 (23.1)	


*^∗^Chi-2 test.*

### *mtSSU rRNA* Target Is Detected in *mtLSU rRNA* Negative Samples

Since *mtSSU rRNA* was demonstrated to be present with a higher number of copies compared to *mtLSU rRNA*, with a 2.5 times more quantification compared to *mtLSU rRNA* (37/15 copies; **Figure 3A**), we tested a second cohort of negative-*mtLSU rRNA* PCR samples for *mtSSU rRNA* amplification. Three samples (two patients) out of 95 were positive for *mtSSU rRNA* amplification. Two of them were a bronchial aspirate (Cq: 34.69) and a BAL fluid (Cq: 33.50) both for the same patient, whereas the remaining one was a induced-sputum (Cq: 33.13) for a patient with a BAL positive for *mtLSU rRNA* amplification but with less than 10 trophic form equivalents/mL (4.5 TfEq/mL). These patients were given anti-*P. jirovecii* prophylaxis after the clinical episode and did not develop PCP within the next 2 years.

## Discussion

PCR assays are now considered as the most useful tool for pneumocystosis diagnosis, especially in HIV-negative immunocompromised patients, with an excellent negative predictive value and a high level of suspicion when PCR is positive (Alanio et al., 2016b). The most commonly used PCR assay is based on the amplification of mitochondrial large subunit ribosomal gene (*mtLSU* rRNA). This target has been proposed and used for 25 years (Wakefield et al., 1990; Meliani et al., 2003; Aderaye et al., 2008; Alanio et al., 2011; Hauser et al., 2011; Botterel et al., 2012). Multicopy genes have been shown to lead to increased detection of *P. jirovecii*. Indeed, *mtLSU* (Montesinos et al., 2015) and *MSG* (Linssen et al., 2006) PCR assays gave increased sensitivity compared to unicopy genes (*DHPS* or β*-tubulin*). However, strict comparison of *mtLSU* and unique gene PCR assays had not been performed until now. In our study, we designed and optimized six PCR assays including four mitochondrial genes assays [*mtLSU* (Alanio et al., 2011), *mtSSU*, *NAD1*, and *CYTB*] and two unique nuclear gene assays (*DHPS*, *HSP70*), with the initial idea to evaluate the number of mitochondrial DNA copies compared to nuclear unique genes. Primer concentration, buffer from different suppliers, and PCR cycles protocols were optimized (see **Supplementary Figures S2**–**S5**) and efficiencies calculated to allow accurate copy number calculations following accurate calculations (Pfaffl, 2001). We then screened a collection of 84 BALs selected to harbor more than 10 EqTr/ml using *mtLSU* PCR assay to increase the chance to get amplification for nuclear unique genes.

The result of the immunofluorescence (IF) assay (positive vs. negative), the PCR fungal load (high vs. medium/low) and the final clinical diagnosis of the episode based on electronic file of the patient’s episode [pneumocystosis (PCP), vs. carriage (PCC)] were first analyzed regarding that of our six PCR assays. As observed before in literature (Mühlethaler et al., 2012), we observed a large overlap in terms of fungal load between IF+ and IF- samples suggesting that the result the IF assay does not reflect perfectly the fungal load in respiratory samples. This can be explained by intrinsic technical issues of IF assay (limited amount of material spotted on slices, variability of the quality of the samples, restriction at ≥3 elements for positive results according to manufacturer’s recommendations, non-specificity of the shape of the acsi compared to yeasts). Another explanation is given by the potential differences in the trophic forms/asci ratio known as about 1:10 in HIV positive patients with PCP and also known to vary upon drug exposure (Tamburrini et al., 1996). If the generation of acsi is impaired for some reason, PCR assays will detect more DNA from trophic forms than from the acsi that would have been missed by the IF assay. This could explain why in our BAL samples about 34–54% of the samples were included in the overlap between the first IF- and the last IF+ samples.

We also found that the final clinical diagnosis as retained by the clinician was not linked to the fungal load. Indeed, we found patients harboring the same fungal load classified as PCP or PCC. No significant difference was observed between fungal loads of PCP patients between HIV+ and HIV- patients although differences were significant when fungal load or IF was considered, which is consistent with literature (Alanio et al., 2011; Botterel et al., 2012; Louis et al., 2015). It is interesting to consider that the clinical decision is not based only on the result of biological tests but include also a broader view including pretest probability, radiological findings, background of the patient, initial evolution, occurrence of co-pathogens, experience of the clinician (Islam et al., 2015). Indeed, four out of five HIV+ patients with CD4 counts <200/mm3 were classified as PCP with IF- and a medium/low fungal load. This clinical decision is coherent with the fact that *P. jirovecii* found in an immunocompromised host even with a medium or low fungal load should be considered carefully in terms of treatment, as recommended in non-HIV immunocompromised patients (Alanio et al., 2016b). Given these observations, it appears that the fungal load would be the most objective criteria for further analysis that prevent any bias due to technical or clinical interpretation.

Using our assays, we observed intriguingly that the number of copies for four mitochondrial genes were different in all the 84 samples, although for the 23 samples from cluster 7, the difference was significant but less noticeable (**Figures 3B,C**). Mitochondrial DNA (mtDNA) is supposed to be circular in *P. jirovecii* as opposed to *P. carinii* or *P. murina* for which a linear structure have been proposed (Ma et al., 2013) and so, each gene is supposed to be present once per mtDNA copies and harboring the same number of copies compared to nuclear genes. Interestingly, *mtLSU rRNA* (median of 15 copies per nuclear genome) and *CYTB* (median of 6 copies per nuclear genome) assays gave less copy number than *NAD1* (median of 23 copies per nuclear genome) or *mtSSU rRNA* (median of 37 copies per nuclear genome).

**FIGURE 5:**
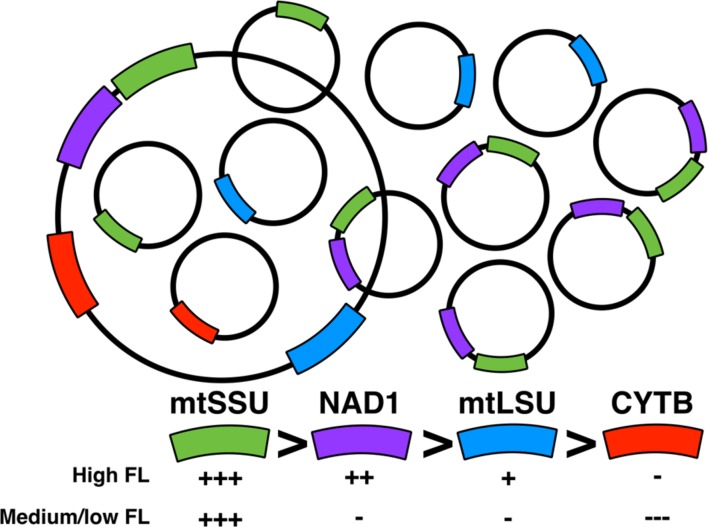
**Model for the dynamics of *P. jirovecii* mtDNA.** High number of mtDNA subspecies is found together with the whole mtDNA species with variation of different subspecies according to the physiology of *P. jirovecii* (fungal load). A subspecies containing *mtSSU* and *NAD1* is present with a higher number copy compared to other mtDNA subspecies.

Our hypothesis is that in *P. jirovecii*, several mtDNA ‘species’ coexists with some of them amplified more than others (**Figure 5**). This is supported by the fact that the physical mapping of the mitochondrial genes [**Supplementary Figure S1**, (Ma et al., 2013)] showed that *NAD1* and *mtSSU rRNA* are closer together than with the other genes. Indeed, one can imagine that a mtDNA subspecies including *NAD1* and *mtSSU rRNA* could be overrepresented compared to other subspecies and compared to the whole mtDNA. The technique used to describe the sequence of *P. jirovecii* was Sanger sequencing from conserved mtDNA regions of *P. murina* and *P. carinii* after cloning and endpoint PCR (Ma et al., 2013). Indeed, this method does not accurately detect subspecies of mtDNA but is accurate to determine the synteny of mitochondrial genes when at least one entire copy of the mtDNA is present in the sample. This observation needs to be analyzed in parallel with another observation we did recently using other samples on mitochondrial heteroplasmy and mtDNA recombination in *P. jirovecii* (Alanio et al., 2016a). Unfortunately, we were not able to validate this hypothesis using endpoint PCR. This can be explained by the fact that large PCR amplicons (>2 kbp) are difficult to obtain in complex DNA extracts as BAL fluids or by the fact that these subspecies are linear preventing amplification of the remaining DNA part that could have been amplified if it is circular.

Interestingly, we found that the number of copies of the different genes vary regarding the fungal load with discrimination between high, medium and low fungal loads. This supports the hypothesis of differential amplification of some subspecies of mtDNA regarding the metabolism state of *P. jirovecii*. A high fungal load would be related to highly proliferating organisms whereas medium/low fungal loads to a less proliferating or a more quiescent state. One can imagine that the physiology of mitochondria would be different in these two situations as already suggested in *Cryptococcus neoformans* (Alanio et al., 2015; Rocheteau et al., 2015), with the differential amplification of some subspecies in these different biological situations, as observed in our study.

In humans, deletion of mitochondrial genes, described as mtDNA depletion syndromes, leads to severe diseases with poor prognosis by impaired energy production affecting different organs like brain, muscles, liver, and digestive tract (El-Hattab and Scaglia, 2013). However, for *P. jirovecii*, alteration of mitochondrial metabolism should not lead to such extremity, due to its adaptation to host parasitism at the surface of the human alveoli. *P. jirovecii* has been shown to uptake from host several factors it is unable to synthesize and the proteins involved in mitochondrial metabolisms should be one of these factors (Ma et al., 2016). Indeed, *P. jirovecii* has lost synthetic pathways such as coenzyme A, thiamine, Glyoxylate, gluconeogenesis, and fermentation, pathways in which mitochondria plays a role, with uptake from lung as a mean to obtain these metabolites (Ma et al., 2016).

We also found that *mtSSU rRNA* is constantly present with a higher number of copies compared to *mtLSU rRNA* with a median ratio of 2.5 copies. This finding suggests that *mtSSU rRNA* could be present twice in the *P. jirovecii* mtDNA. In addition, *mtSSU rRNA* was demonstrated to be a more sensitive target to detect *P. jirovecii* in respiratory samples since some *mtLSU* negative/*mtSSU* positive samples were found in our collection. This is of prime importance since immunocompromised patients with a positive *P. jirovecii* PCR in respiratory samples would benefit from a prophylactic treatment since detection of *P. jirovecii* at low fungal load could be the first step before the development of pneumocystosis (Mori et al., 2009). Interestingly, *mtSSU rRNA* is the most stable mitochondrial marker regarding fungal load, immunofluorescence results or final clinical diagnosis. All these data suggest that *mtSSU* PCR assay is the best PCR assay to detect *P. jirovecii* in humans. To conclude, further studies on *P. jirovecii* mitochondria are highly needed to validate our hypothesis suggesting plasticity of *P. jirovecii* mitochondrial genome (increased or decreased copy number) and to validate hypotheses from our previous work suggesting heteroplasmy and mtDNA recombination (Alanio et al., 2016a), but its unculturable feature prevents the easy implementation of such studies.

## Author Contributions

AA and MJB conceived and designed the experiments. CV, MG-M, MB, AS-L performed the experiments. AA and CV analyzed the data. CV and AA wrote the manuscript. SB, MJB, SH, and NG reviewed the manuscript. All authors read and approved the final manuscript.

## Conflict of Interest Statement

The authors declare that the research was conducted in the absence of any commercial or financial relationships that could be construed as a potential conflict of interest.

## Supplementary Material

**FIGURE S1 | Genetic map of *Pneumocystis jirovecii* mtDNA based on JX499143 sequence is shown in linear configuration.** *mtSSU rRNA*, *mtLSU rRNA*, *NAD1*, and *CYTB* genes are shown emphasized within the figure.

**FIGURE S2 | Optimization of qPCR assays I.** *mtLSU rRNA* gene (top of the figure) was tested with Roche mastermix (LightCycler 480 Probes Master, Roche Diagnostics, Mannheim, Germany) and 0.3 μM of each primer and 0.1 μM of probe (top left panel), 0.5 μM of each primer and 0.25 μM of probe (top right panel), 0.6 μM of each primer and 0.3 μM of probe (lower left panel) and 0.3 μM of each primer and 0.1 μM of probe again but with Applied mastermix (TaqMan Universal PCR Master Mix, Applied Biosystems, Carlsbad, CA, USA) (lower right panel). *mtSSU rRNA* gene (beneath of the figure) was tested with 0.3 μM of each primer and 0.1 μM of probe with Roche (left panel) and Applied (right panel) matermixes. Cq values obtained for each dilution of positive control DNA as well as efficiency calculated with those data are represented in a table next to the panels. Optimal PCR conditions are highlighted in color.

**FIGURE S3 | Optimization of qPCR assays II.** *CYTB* gene (top of the figure) was optimized by testing Roche mastermix with 0.3 μM of each primer and 0.1 μM of probe (top left panel), 0.5 μM of each primer and 0.25 μM of probe (top right panel), 0.6 μM of each primer and 0.3 μM of probe (lower left panel) and 0.3 μM of each primer and 0.1 μM of probe again but with Applied mastermix (lower right panel). *NAD1* gene (beneath of the figure) was tested with 0.3 μM of each primer and 0.1 μM of probe with Roche (left panel) and Applied (right panel) matermixes. Cq values obtained for each dilution of positive control DNA as well as efficiency calculated with those data are represented in a table next to the panels. Optimal PCR conditions are highlighted in color.

**FIGURE S4 | Optimization of qPCR assays III.** *DHPS* gene (top of the figure) was optimized by testing Roche mastermix with 0.3 μM of each primer and 0.1 μM of probe (top left panel), 0.5 μM of each primer and 0.25 μM of probe (top right panel), 0.6 μM of each primer and 0.3 μM of probe (lower left panel) and 0.3 μM of each primer and 0.1 μM of probe again but with Applied mastermix (lower right panel). *HSP70* gene (beneath of the figure) was tested with 0.3 μM of each primer and 0.1 μM of probe with Roche (left panel) and Applied (right panel) matermixes. Cq values obtained for each dilution of positive control DNA as well as efficiency calculated with those data are represented in a table next to the panels. Optimal PCR conditions are highlighted in color.

**FIGURE S5 | Optimization of qPCR assays IV. Optimal PCR conditions are shown at once.**

**FIGURE S6 | *Pneumocystis jirovecii* quantification regarding clinical background of patients.** *NAD1* quantification in high fungal load samples (left) and medium/low fungal load (right) **(A)**. *CYTB* quantification in high fungal load samples (left) and medium/low fungal load (right) **(B)**. *mtLSU rRNA* quantification in PCP samples (left) and PCC (right) **(C)**. Nuclear genes quantification PCP samples (left) and PCC (right) **(D)**.

**FIGURE S7 | Copy number variation of mitochondrial genes varies according to the fungal load.** CNV is significantly different in samples with high, medium and low fungal loads for *CYTB* and *NAD1* but not for *mtLSU* or *mtSSU* **(A)**. All ratio of the DNA of the four mitochondrial gene is significantly different in samples with high medium or low fungal loads **(B)**. *P-*values indicating the significance of the differences are indicated in the top of the figure for each category.

**FIGURE S8 | Copy number variation of mitochondrial genes do not vary according to immunofluorescence results and final clinical diagnosis.** CNV of mitochondrial genes in IF+ and IF- samples **(A)**. CNV in ratios between these mitochondrial genes in IF+ and IF- samples **(B)**. CNV of mitochondrial genes in PCP and PCC samples **(C)** CNV in ratios between these mitochondrial genes in PCP and PCC samples **(D)**. *P-*values indicating the significance of the differences are indicated in the top of the figure for each category.

**Table S1 | Sequences and targets of primers and probes used in *P. jirovecii* quantification assays and their corresponding amplicon sizes.**
