# Supplementary figures and images for: Copy Number Variation of Mitochondrial DNA Genes in Pneumocystis jirovecii According to the Fungal Load in BAL Specimens
Source: Front Microbiol. 2016 Sep 12;7:1413. doi: 10.3389/fmicb.2016.01413 (PMC5018473; doi:10.3389/fmicb.2016.01413)

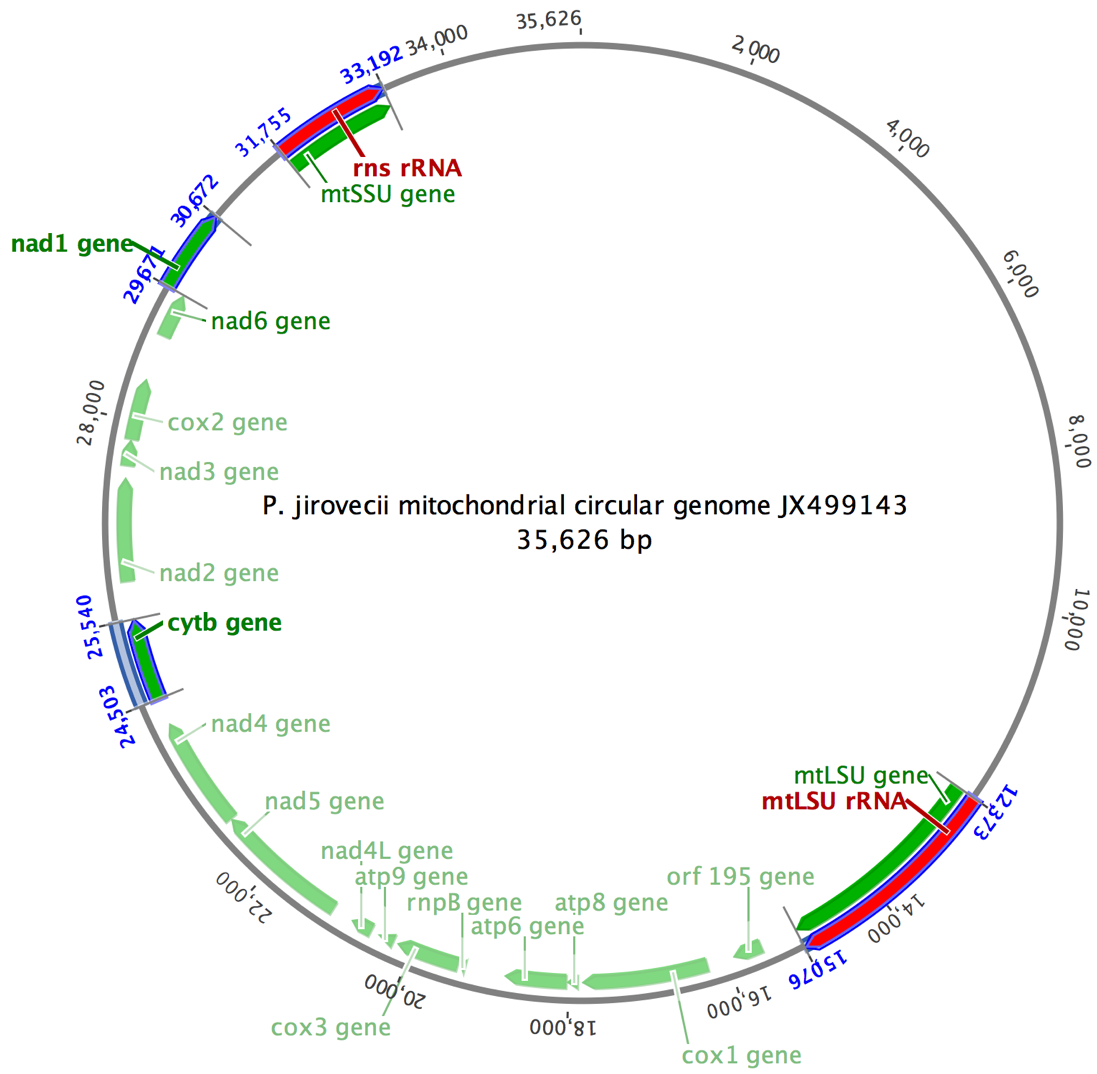

Supplement: Supplementary file 1 [file Image_1.TIFF]

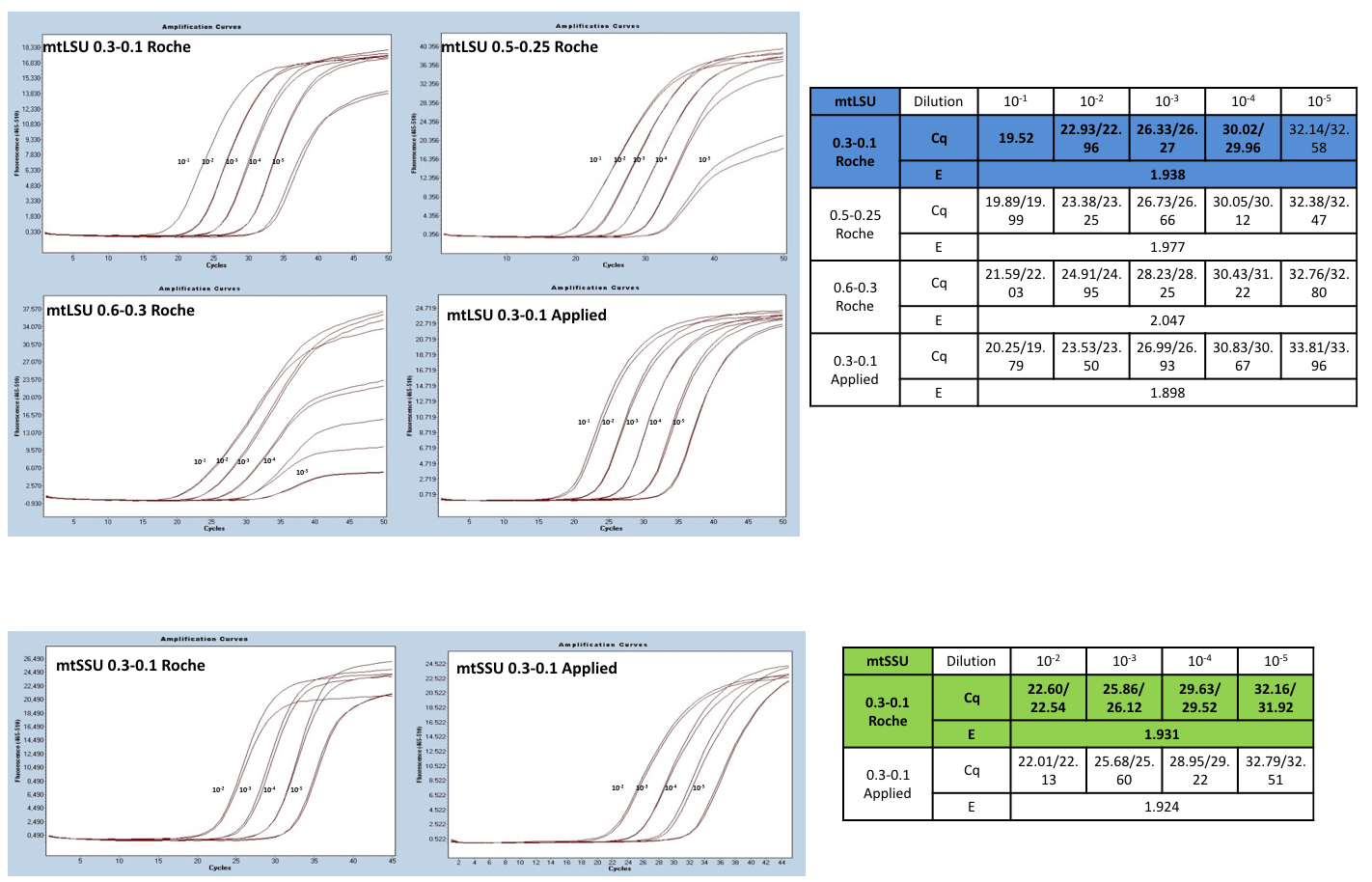

Supplement: Supplementary file 2 [file Image_2.TIFF]

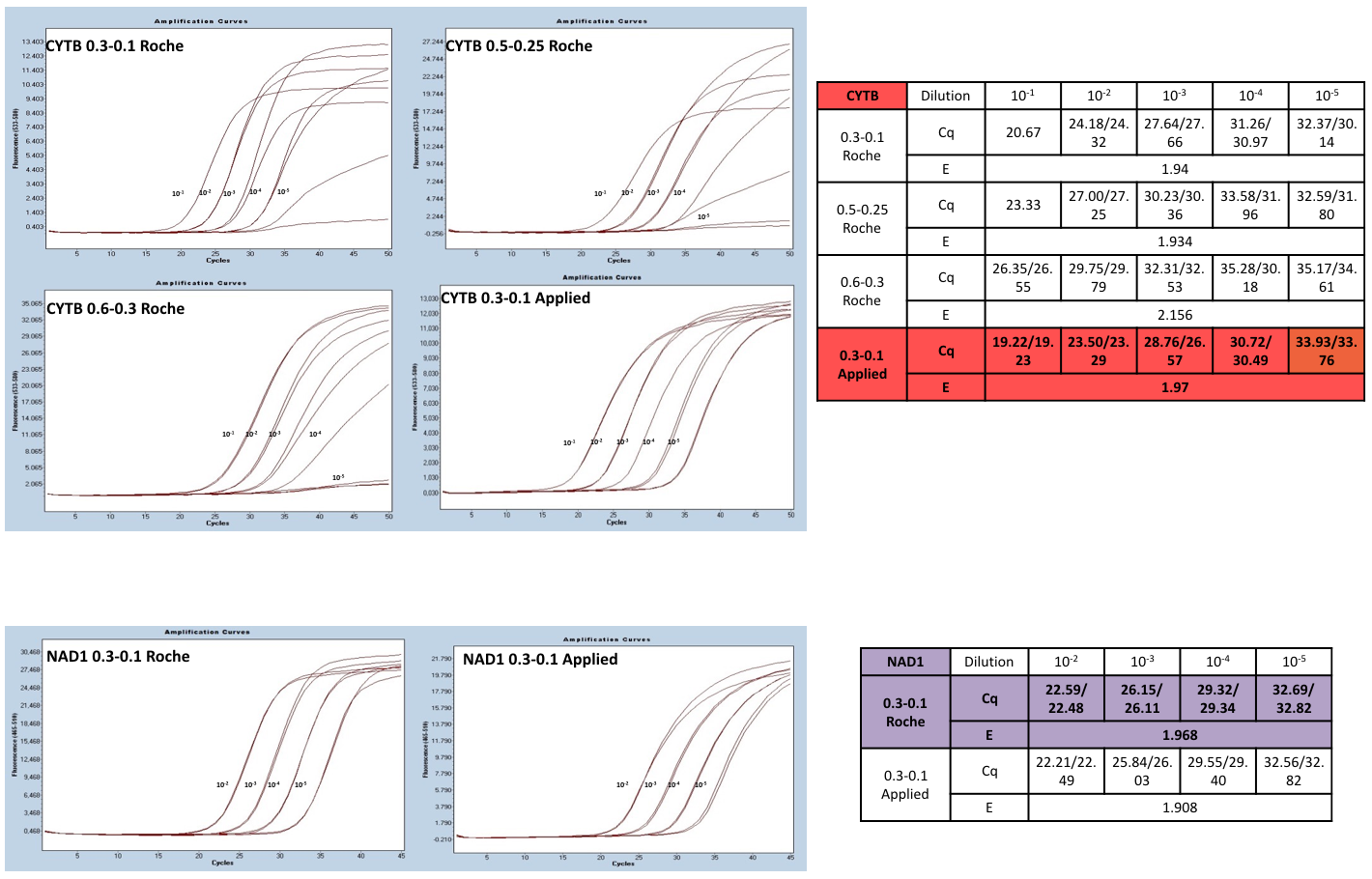

Supplement: Supplementary file 3 [file Image_3.TIFF]

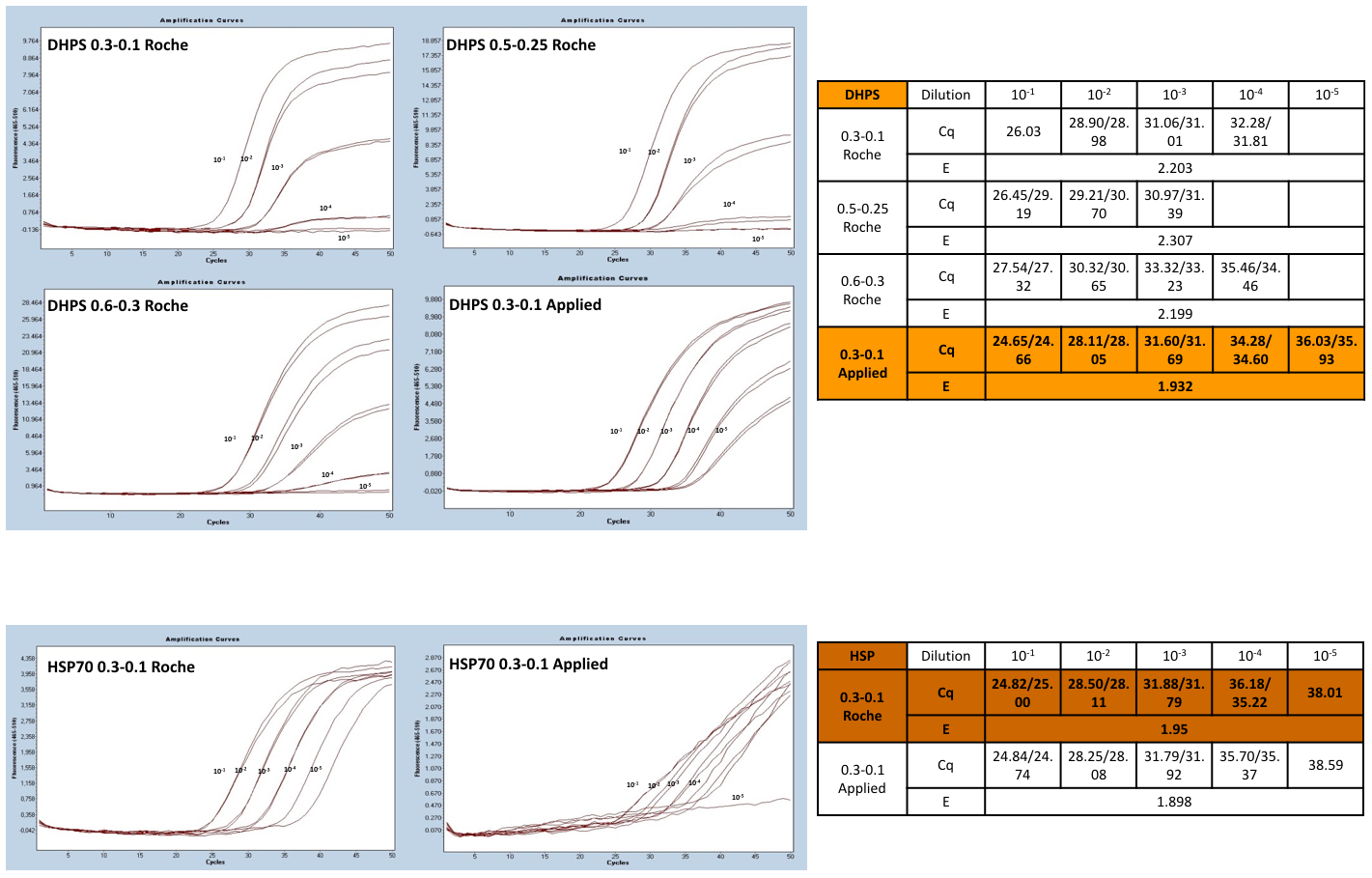

Supplement: Supplementary file 4 [file Image_4.TIFF]

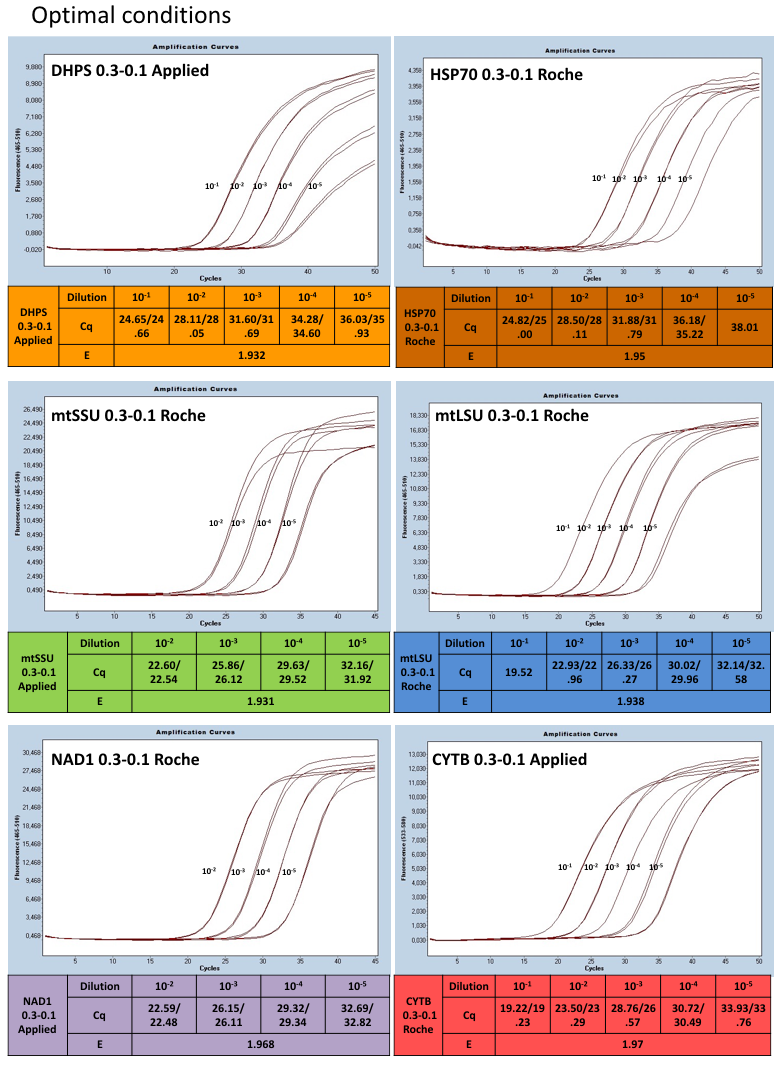

Supplement: Supplementary file 5 [file Image_5.TIFF]

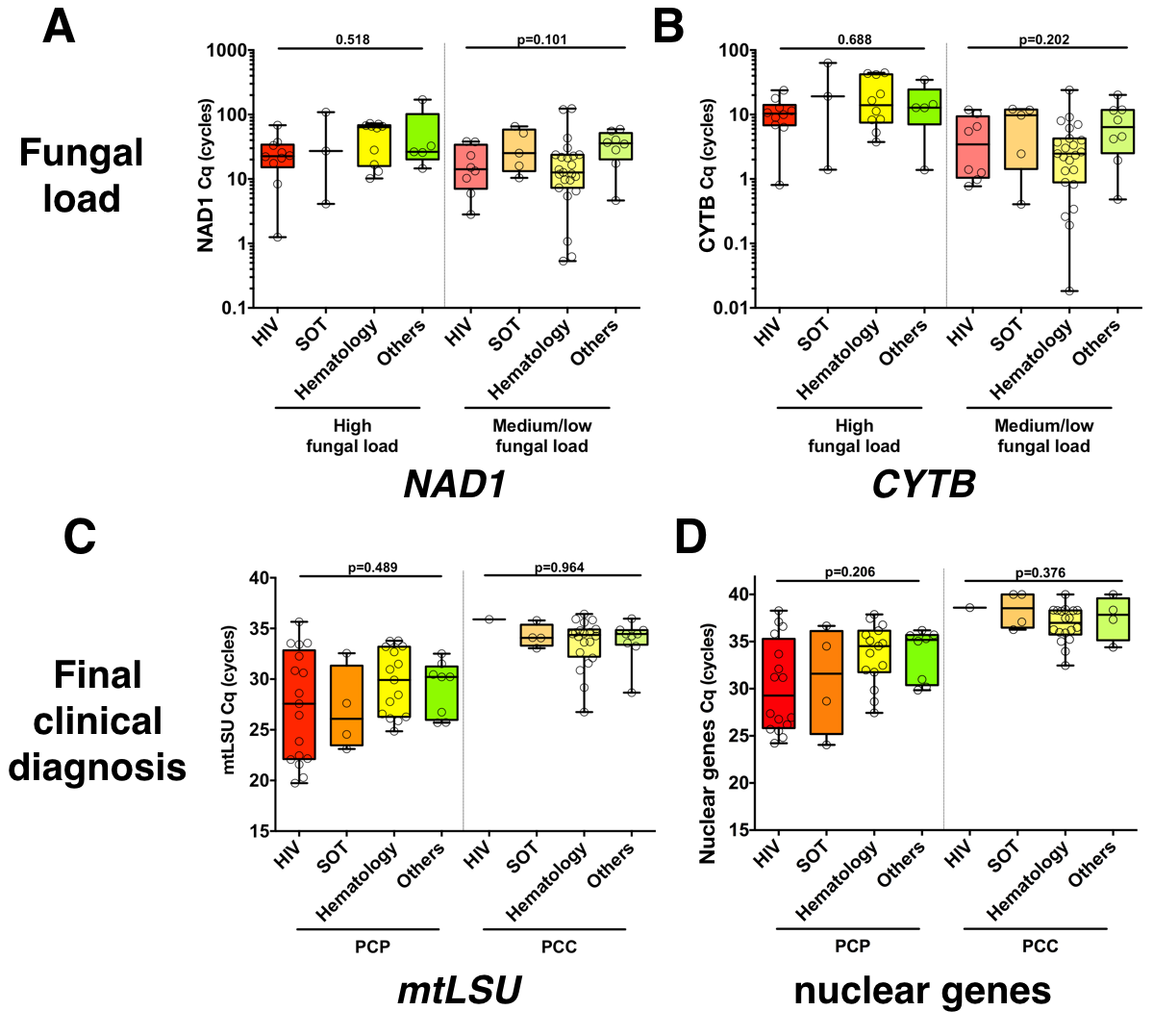

Supplement: Supplementary file 6 [file Image_6.TIFF]

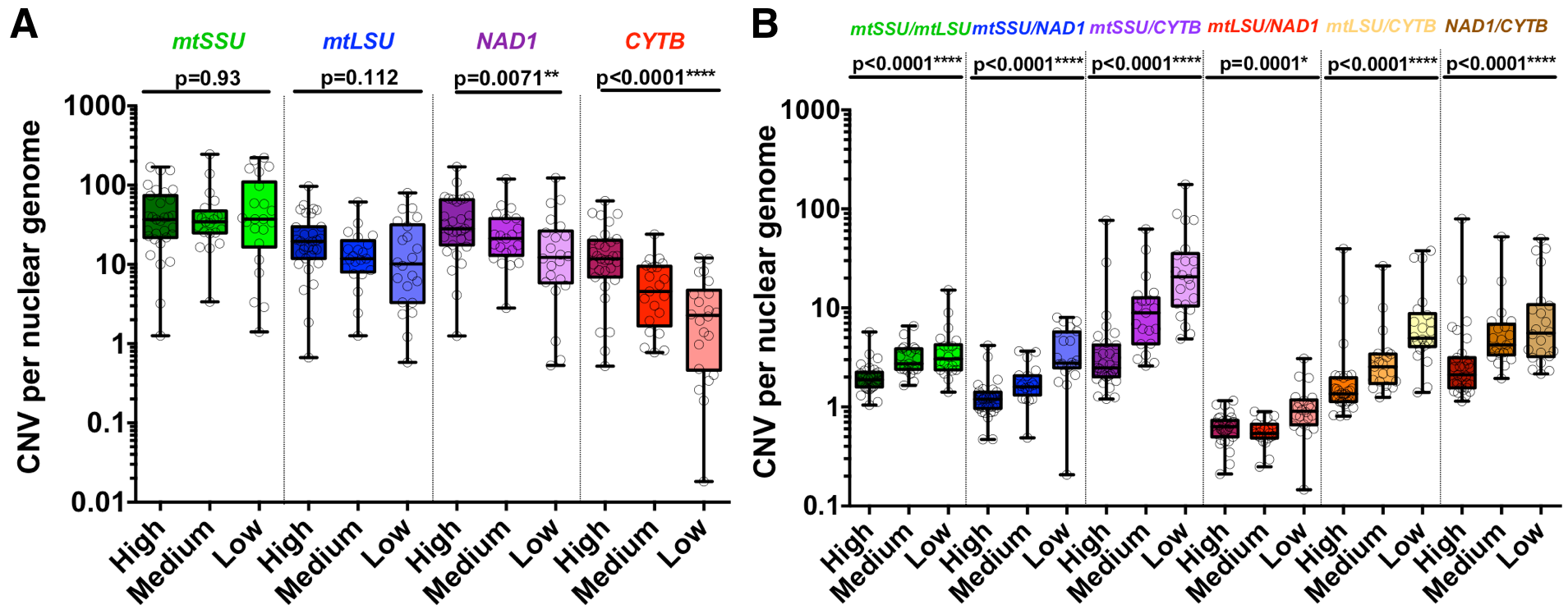

Supplement: Supplementary file 7 [file Image_7.TIFF]

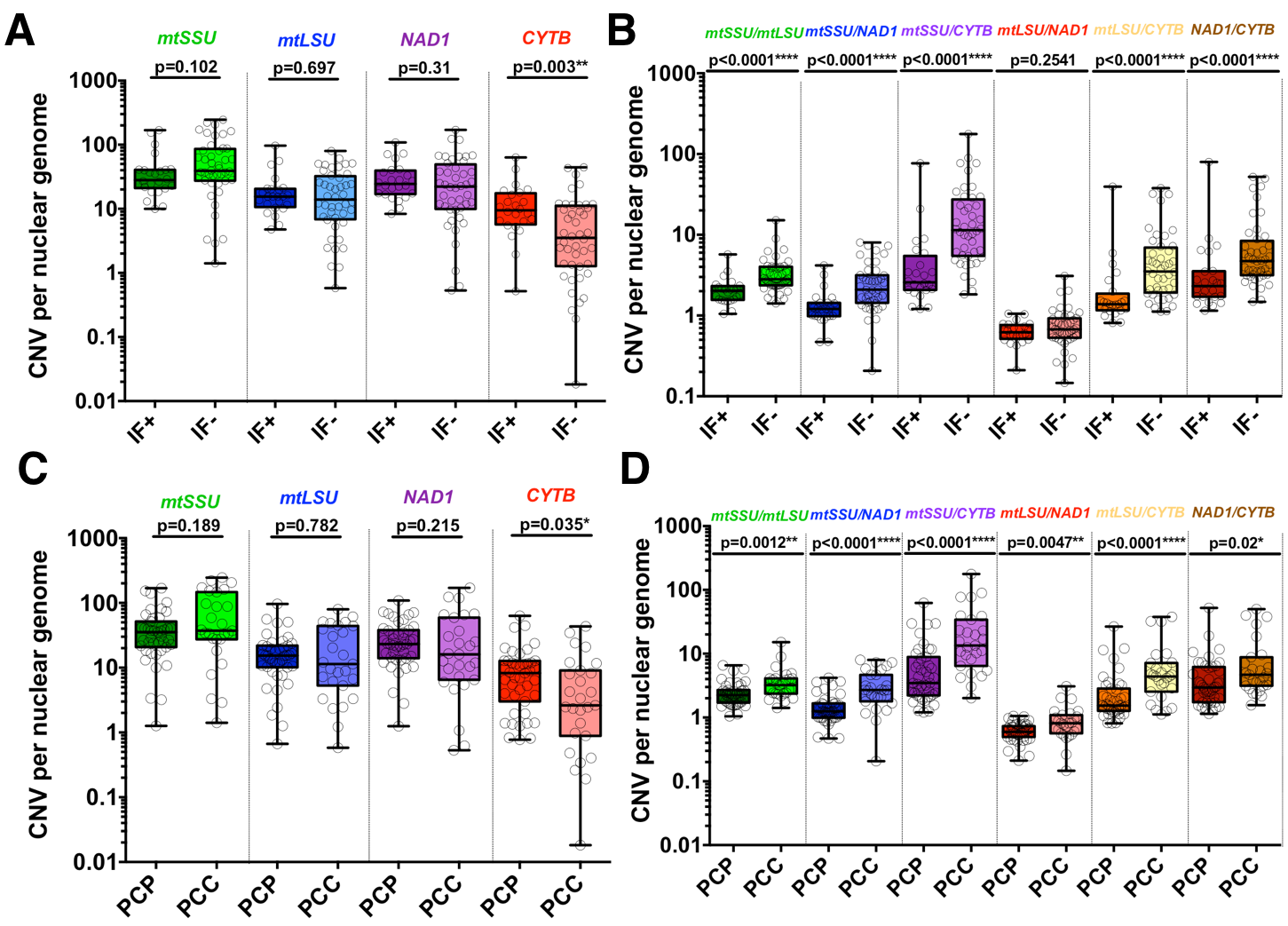

Supplement: Supplementary file 8 [file Image_8.TIFF]
