# Supplementary material for: Copy Number Variation of Mitochondrial DNA Genes in Pneumocystis jirovecii According to the Fungal Load in BAL Specimens
Source: Front Microbiol. 2016 Sep 12;7:1413. doi: 10.3389/fmicb.2016.01413 (PMC5018473; doi:10.3389/fmicb.2016.01413)
Supplement: Supplementary file 9 [file Table_1.DOCX]

Table S1: Sequences and targets of primers and probes used in *P. jirovecii* quantification assays and their corresponding amplicon sizes

| **PRIMERS AND PROBES** | **TARGET** | **SEQUENCE** | **Amplicon size (bp)** |
| --- | --- | --- | --- |
| mtSSU_Pj1098F (f) |  | 5’-TCATGACCCTTATGAAGTGGGC-3’ |  |
| mtSSU_Pj1173R (r) | *mtSSU rRNA* | 5’-GCTCCGACTTCCATCATTGC-3’ | 76 |
| mtSSU_P1125P |  | 5’-**FAM**-ACGTGCTGCAAAATTTTCTACAATGGG-BHQ1-3’ |  |
| PjF1 (f) | *mtLSU rRNA* | 5’-CTGTTTCCCTTTCGACTATCTACCTT-3’ | 121 |
| PjR1 (r) |  | 5’-CACTGAATATCTCGAGGGAGTATGAA-3’ |  |
| PjSL |  | 5’-**FAM**-TCGCACATAGTCTGATTAT- MGB-3’ |  |
| NAD1_Pj579F (f) | *NAD1* | 5’-AGCAGAAACGAATTGAGCTCCT-3’ | 86 |
| NAD1_Pj664R (r) |  | 5’-TCGCAGCAGAATACTCAGTCAT-3’ |  |
| NAD1_Pj608P |  | 5’-**FAM**-TGCCAGAAGCTGAATCCGAATTAGTTGC-BHQ1-3’ |  |
| CYTB_Pj241F (f) | *CYTB* | 5’-ACTCCCAGAATTCTCGTTTGGT-3’ | 112 |
| CYTB_Pj352R (r) |  | 5’-TCGCTCCCCACAATGACATT-3’ |  |
| CYTB_Pj302P |  | 5’-**HEX**-CTTTCTTGGGATATGTTCTGCC-BHQ1-3’ |  |
| DHPS-F (f) | *DHPS* | 5’-GTTTGCCTTGGTTGCTTGGT-3’ | 93 |
| DHPS-R (r) |  | 5’-CAGCAGTGCCCCAAATCCT-3’ |  |
| DHPS-probe |  | 5’-**FAM**-AGATTTACAGGGTGTCTTAC- MGB-3’ |  |
| HSP70_Pj126F (f) | *HSP70* | 5’-GGAGATTTCATCAATGGTCCTT -3’ | 77 |
| HSP70_Pj202R (r) |  | 5’-CGGCATTGGAAACTTTAGTCC -3’ |  |
| HSP70_Pj157P |  | 5’-**FAM**-AAGGAGGTGGCAGAAGCGTA- BHQ1-3’ |  |

f, forward; r, reverse; BHQ1, black hole quencher 1; MGB, Minor Groove Binder; Probe fluorophores are detailed in bold type.s
